# Benign metastasizing uterine leiomyoma with lymphatic and pulmonary metastases: a case report and literature review

**DOI:** 10.1186/s12905-023-02237-y

**Published:** 2023-04-01

**Authors:** Tong Tong, Qiong Fan, Yudong Wang, Yuhong Li

**Affiliations:** Department of Gynecological Oncology, The International Peace Maternity and Child Health Hospital, School of Medicine, Shanghai Jiao Tong University, Hengshan Road No. 910, Shanghai, 200030 China

**Keywords:** Benign metastasizing leiomyoma, Lymph node, Pulmonary metastases, Case report

## Abstract

**Background:**

Benign metastasizing leiomyoma (BML) is a rare disease usually observed in women of reproductive or premenopausal age with a history of uterine myomectomy or hysterectomy. The most common sites of metastases are the pulmonary, and other sites include heart, bones, liver, lymph nodes, bladder, skeletal muscles, and central nervous system. Here, we report a case of a 50 year-old woman with a history of hysterectomy who was initially suspected of uterine sarcoma but was finally confirmed to have BML with lung and lymph node metastases, and discuss the treatment and prognosis of BML.

**Case presentation:**

A 50 year-old woman with a history of total abdominal hysterectomy presented with mild but persistent abdominal pain for more than 3 months. She was suspected of having uterine sarcoma before surgery and laparoscopic extensive debulking surgery including bilateral oophorectomy, pelvic and para-aortic lymph node dissection to the level of the left renal vein, and transcutaneous dissection of the right inguinal lymph nodes. Pathology confirmed a benign leiomyoma, and the patient was diagnosed with BML. No medication was administered after the surgery, and the follow-up was of no significance.

**Conclusion:**

Benign metastasizing leiomyoma (BML) is a rare disorder in which histologically benign smooth muscle tumors metastasize to extrauterine sites. Metastases are commonly observed in the lung, liver, lymph nodes, skin, bladder, esophagus, and skeletal muscles. BML is usually misdiagnosed as a malignant tumor before surgery until the pathology confirms its benign nature. However, this treatment remains controversial and undetermined. The prognosis is usually favorable owing to its benign nature.

## Background

Benign metastasizing leiomyoma (BML) is a rare disorder that occurs mainly in women of reproductive or premenopausal age with a history of uterine myomectomy or hysterectomy. Histologically, BML is a benign tumor that originates from smooth muscle cells that metastasize to extrauterine sites. The most common sites of metastases are the pulmonary, and other sites include heart, bones, liver, lymph nodes, skin, bladder, esophagus, skeletal muscles, and central nervous system [1–11]. Multiple metastases simultaneously, including lymph node metastases in BML, are extremely rare, and studies on the prognosis and treatment of BML with multiple lymph node metastases are scarce. Here, we report a case of BML with simultaneous pulmonary and lymph node metastases at the same time and discuss the clinical and radiologic features of BML.

## Case presentation

A 50 year-old woman with no significant medical history (parity 2-0-0-2, both normal spontaneous vaginal deliveries) presented with mild but persistent abdominal pain for over 3 months. She underwent abdominal total hysterectomy and bilateral salpingectomy for uterine leiomyoma at a local secondary hospital in January 2019. Pathological examination revealed benign leiomyoma. The post-operative Regular follow-up was not significant. However, she experienced persistent lower abdominal pain in the past 3 months but did not suffer from weight loss or any other respiratory symptoms such as hemoptysis, purulent sputum, or exertional dyspnea. Ultrasound at a local clinic revealed a heterogeneous solid mass measuring 4 cm in diameter in the central pelvis. She was referred to the International Peace Maternity and Child Health Hospital for further work.

**Fig. 1:**
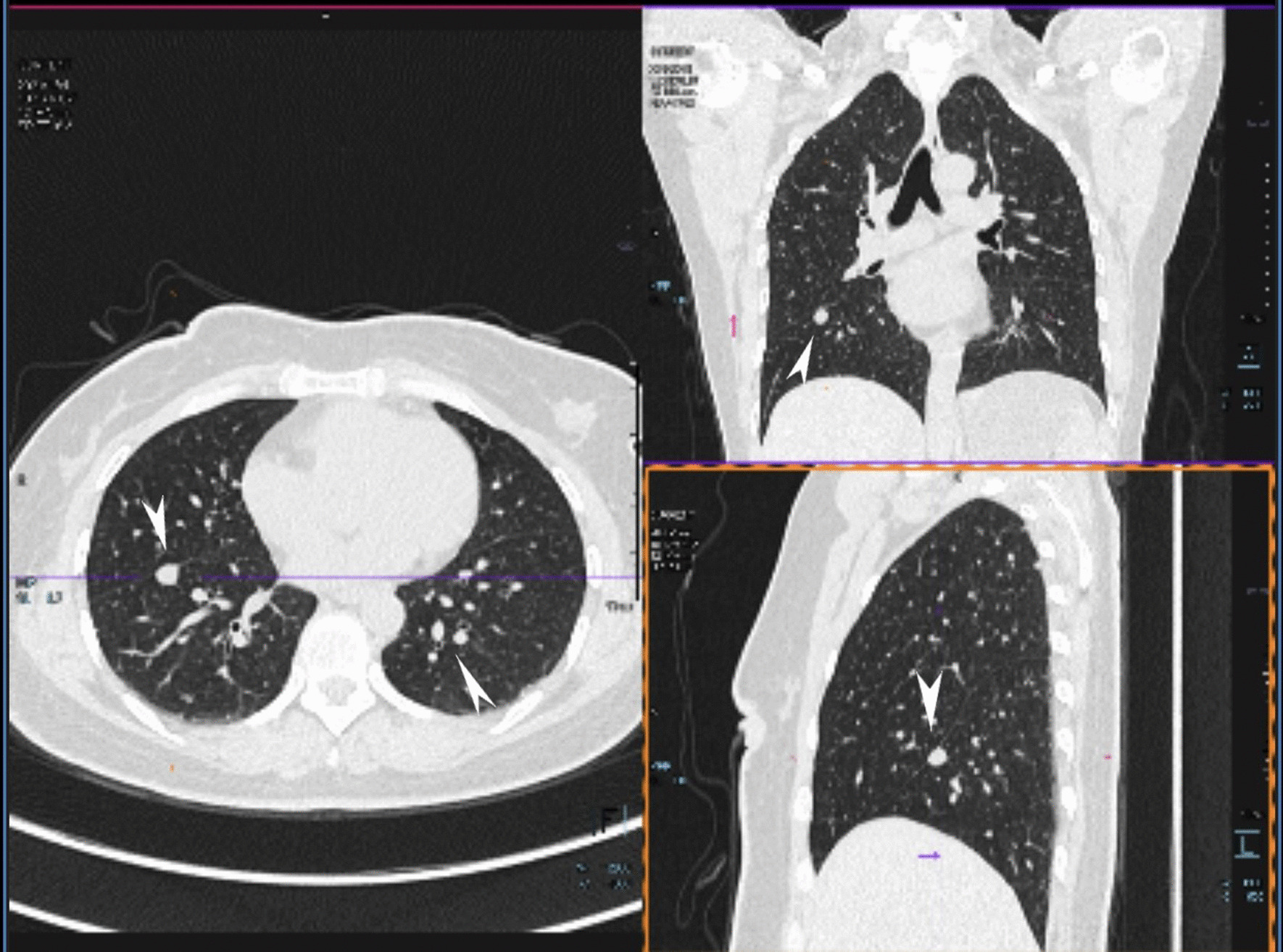
Multiple metastasis in the bilateral lungs (see arrow)

**Fig. 2:**
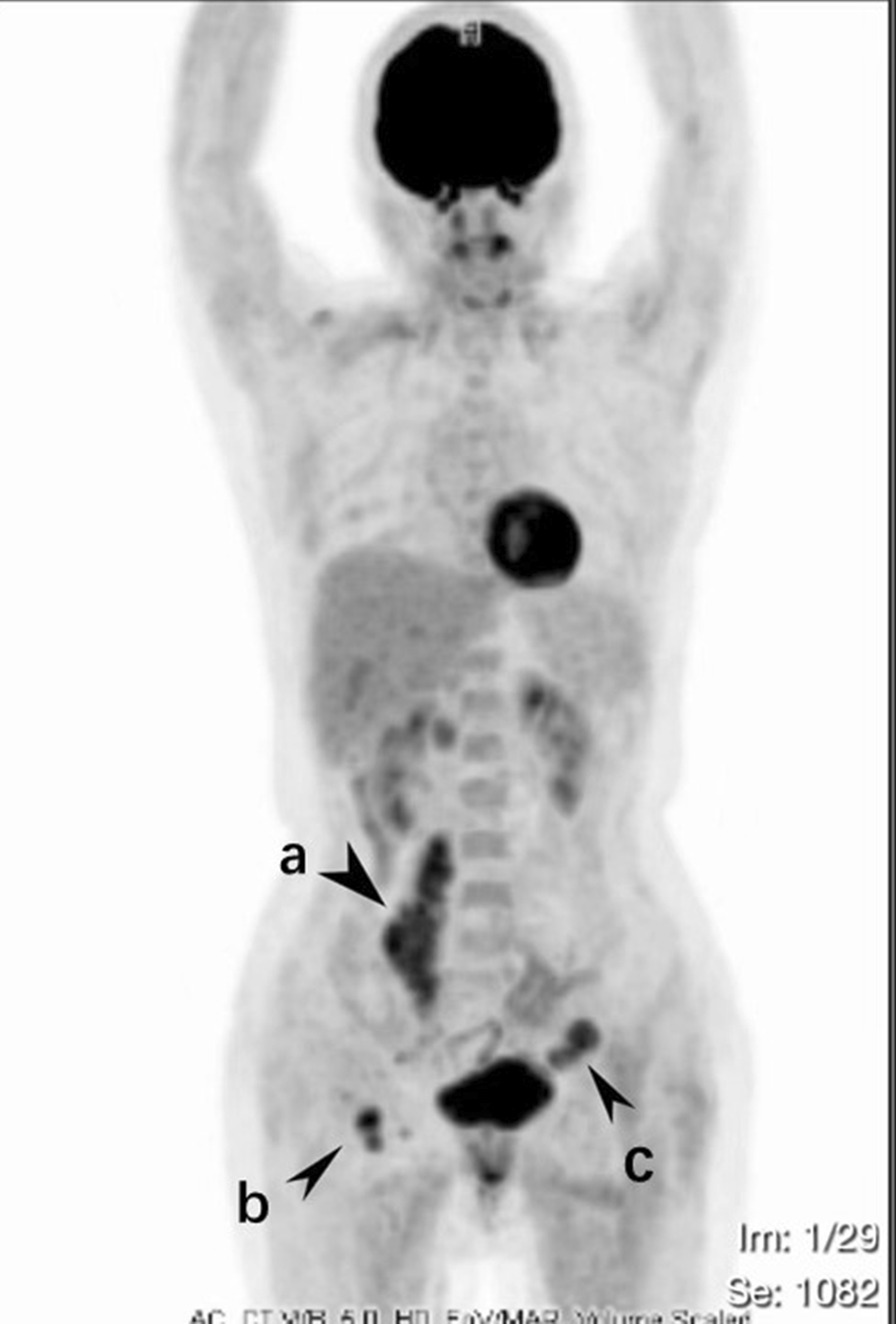
Multiple metastasis in pelvic, along post cava and the right inguinal lymph nodes (**a** Tumor along the right pelvic infundibular ligament; **b** Retroperitoneal tumor of left pelvic; **c** Enlarged lymph nodes of right inguinal)

**Fig. 3:**
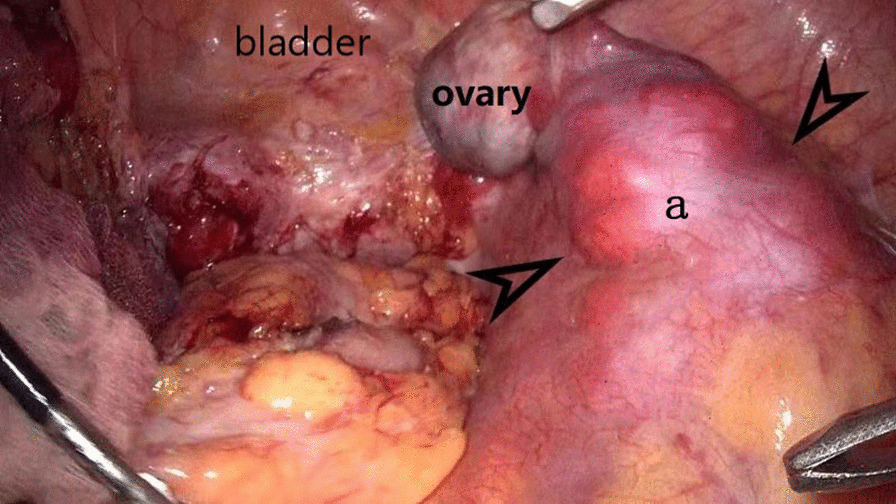
Leiomyoma metastasis along the post cava seen in the laparoscopic surgery (a tumor along the right pelvic infundibular ligament to the level of renal vein)

**Fig. 4:**
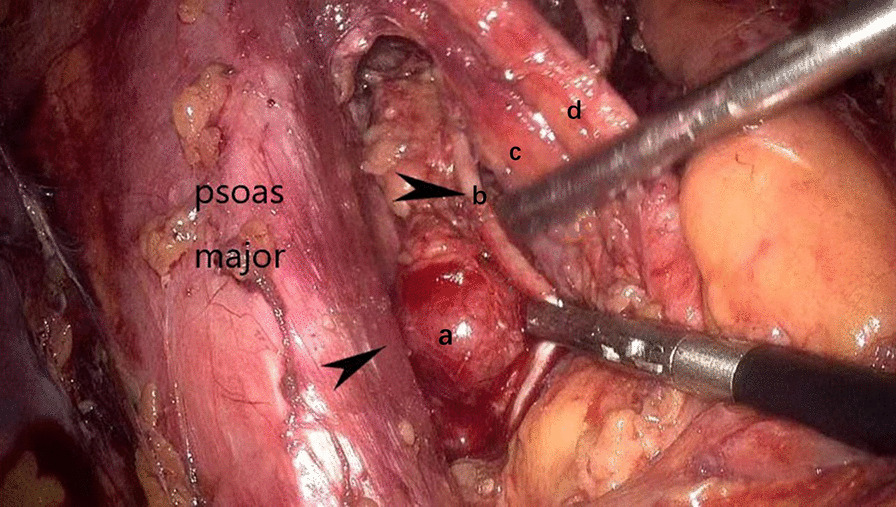
Leiomyoma metastasis to the internal iliac lymph nodes seen in the laparoscopic surgery (**a** Enlarged left internal iliac lymph nodes; **b** Left obturator nerve; **c** Vena iliaca externa; **d** External iliac artery)

**Fig. 5:**
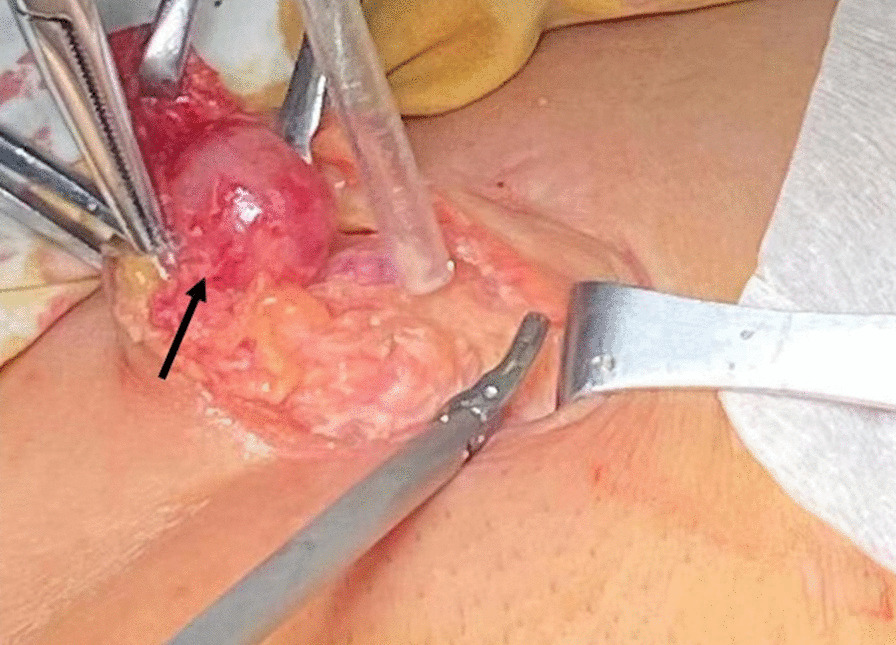
Tumor metastasis to the right inguinal lymph nodes (see arrow)

Routine laboratory tests showed that the values for biochemical variables, including tumor markers such as alpha-fetoprotein, cancer antigen 19‑9, and cancer antigen 125, were within the normal ranges. Pelvic magnetic resonance imaging (MRI) revealed solid pelvic masses in the central and bilateral pelvis, and enlargement of multiple lymph nodes around the iliac chain and aorta. On chest CT, there were multiple well-circumscribed nodules in the bilateral pulmonary arteries, with the largest nodule 1.5 cm in diameter (Fig. 1). An original pathology slide review and positron emission tomography (PET)-CT were done. According to the original slide review, it was a benign leiomyoma with a mitotic count of < 5/10 high-power fields (HPF), little cytological atypia, and no tumor cell necrosis, but with cellular abundance. Abnormal fluorodeoxyglucose (FDG) uptake in the bi-adnexal mass with the suspicion of para-aortic, aorto-caval, precaval, external iliac, and bilateral inguinal lymph node metastases was found on PET-CT (Fig. 2). Multiple nodules in both lungs without abnormal FDG uptake were considered metastatic cancers. An additional chest computed tomography (CT) scan revealed metastatic lung cancer. Considering the patient’s history of gynecologic surgery, pelvic malignancy of unknown origin was suspected. In December 2020, laparoscopic extensive debulking surgery, including bilateral oophorectomy, pelvic and para-aortic lymph node dissection to the level of the left renal vein, and right inguinal lymph node dissection, was performed. Grossly, there were masses 5 cm in size on the left-side stump of the vagina and along the right pelvic infundibular ligament to the level of the renal vein, measuring 10 × 4 cm (Figs. 3 and 4). All lymph nodes stated above, including the para-aortic, aorto-caval, precaval, and pelvic lymph nodes, were enlarged to approximately 0.5–3 cm in size. The right inguinal lymph nodes were palpable, enlarged to 3 cm in size, and dissected (Fig. 5).

**Fig. 6:**
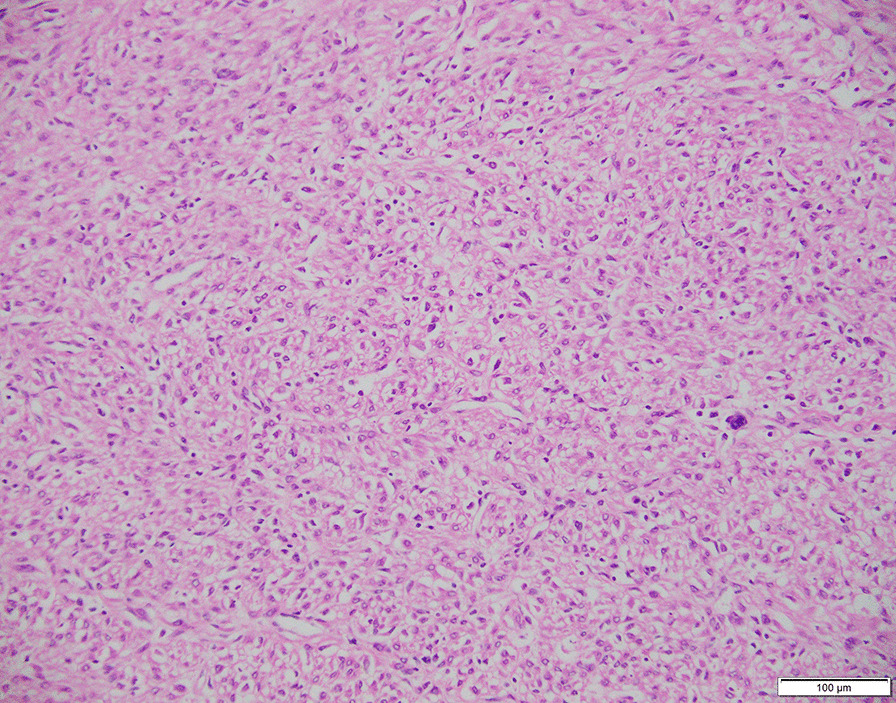
Hematoxylin and eosin (HE) staining of the pelvic tumor specimen

Pathology of the pelvic masses also revealed benign leiomyoma with mitosis < 5/10 HPF, little cytological atypia, and no tumor necrosis, but with cellular abundance and a few singular kernels (Fig. 6). Metastases to the meso-ovarium, bilateral pelvic infundibular ligaments, pelvic lymph nodes, para-aortic lymph nodes, and bilateral inguinal lymph nodes were confirmed in the final pathology report. Metastatic lesions were also reported to have a histology similar to that of the original mass with a mitotic count < 2/10 HPF, little cytological atypism, and no necrosis. On immunohistochemical staining, cells were all positive for estrogen/progesterone receptors, p53, SMA+, HMB45-, Ki67(5%), CD31, and ERG. Clinicopathologically, BML was selected as the final diagnosis after discussion with our pathologists. The patient was discharged from the hospital on post-operative day 7 without any early post-operative complications. No specific treatments were administered. Her current general condition was satisfactory without any postoperative complications. However, long-term follow-up is required to assess disease recurrence and distant metastasis.

## Discussion and conclusions

Benign metastasizing leiomyoma (BML) is a rare disorder in which histologically benign smooth muscle tumors metastasize to extrauterine sites [12]. In 1939 Steiner [13] first reported it as a fibroleiomyomatous hamartoma. Approximately 150 cases of BML have been reported in literature.

The disease commonly occurs in women of late reproductive or premenopausal age, most of whom have a history of leiomyomas treated surgically with myomectomy or hysterectomy [14]. The most frequent site of metastasis was the lungs (80%). Metastases to other organs reported to date include the heart (approximately 10 cases), liver, lymph nodes, skeletal muscle, skin, esophagus, and central nervous system. Multiple metastases, including to lymph nodes, are extremely rare, as reported in this case. BML with lymph node metastasis reported in the literature are shown in Table 1.

**Table 1:** Summary of BML cases with lymph nodes metastasis

Metastasis	Age	History of gynecologic surgery	Surgical treatment
Lymph nodes only	52 [15]	N/A	Total abdominal hysterectomy, bilateral salpingo-oophorectomy, and lymphadenectomy
	27 [16]	N/A	N/A
	69 [17]	N/A	Complete inguinal lymph node excision
	32 [18]	N/A	N/A
Lymph nodes and biceps	47 [19]	Hysterectomy 2 years earlier	Open laparotomy and extirpation of the abdominal tumors
Lymph nodes/retroperitoneal mass and pulmonary	48 [20]	Myomectomy 20 years earlier and then a total abdominal hysterectomy 8 years later	Retroperitoneal mass excision and bilateral salpingo-oophorectomy
	34 [10]	Abdominal myomectomy 1 year earlier	Extensive debulking surgery including total abdominal hysterectomy, bilateral salpingo-oophorectomy, pelvic and paraaortic lymph node dissection to the level of the left renal vein, and gonadal lymph node dissection
	50 [21]	Cesarean sections 16, 13 and 11 years previously	A total abdominal hysterectomy bilateral salpingo-oophorectomy and a dissection of the pelvic and para-aortic lymph nodes
Multiple metastasis (lung, skin, lymph nodes, bone and perhaps the brain)	55 [22]	14 years after uterine myomectomy	N/A

The pathological origin of BML remains unclear and controversial. Several hypotheses have been proposed: (i) mechanical dissemination or intravascular metastases of smooth muscle cells from the uterus to distant locations, (ii) derivation from multifocal but independent smooth muscle proliferation, and (iii) derivation from low-grade leiomyosarcoma [23, 24]. The most commonly accepted theory is the hematogenous spread of a monoclonal element in a benign smooth muscle tumor. Chromosomal abnormalities can be found in 25% of such tumors, including balanced translocation, trisomy 12, or rearrangement of 6p [25, 26]. Other possible mechanisms include lymphovascular embolization, mesothelial mesenchymal metaplasia, and metastases from misdiagnosed low-grade uterine leiomyosarcomas [27, 28].

Histologically, BML has been mainly associated with leiomyomas; however, the type of leiomyoma is not yet clear (eg. Cellular, hemorrhagic cellular, mitotically active, atypical, myxoid, vascular, epithelioid, or lipoleiomyoma). Some studies suggest that BML is a low-grade endometrial stromal sarcoma or malignant uterine leiomyoma, but the evidence supporting these concepts is not very strong.

On imaging examination, such as CT scans of the lungs, BML present as well-circumscribed nodules in the lungs. Bilateral lung nodules are more commonly observed than multiple unilateral lesions or solitary nodules. The nodules of BML can be large or small and cavitate, which can lead to thin-walled or thick-walled cysts [29].

For PET-CT scans, some authors propose that 18‑FDG‑PET/CT is useful for distinguishing malignant leiomyosarcoma from benign leiomyoma, as a number of reports have demonstrated that there is a lack of 18‑FDG uptake in PBML [30]. The SUV of leiomyosarcoma were significantly greater than those of leiomyoma. However, there are numerous reports of 18‑FDG‑avid leiomyoma. Theoretically, PET-CT with 18F–FDG is helpful in assessing the significant metabolic activity of both pulmonary and extra-pulmonary nodules, which should be unexpected in BML and otherwise would raise concern for malignant disease [31, 32]. As in our case, the PET-CT of the patient showed 18-FDG-avid lymph node metastases and lack of 18-FDG uptake in pulmonary lesions, which should raise the concern for the diagnosis of BML.

The immunohistochemical profile of BML is positive for actin, desmin, and smooth muscle actin and has low Ki-67 expression (< 5%) [33], and is difficult to distinguish from primary uterine leiomyoma. High levels of the tumor suppressor gene p53 have also been found in BML lesions; however, its role in pathogenesis has not been clarified.

In theory, BML is estrogen and progesterone receptor positive, and estrogen is known to stimulate tumor growth, while progesterone is known to inhibit tumor growth [34]. However, in a few cases, BML cells were found to lack estrogen or progesterone receptors, suggesting that the site of origin was not the uterus. Another possible explanation is that these receptors are downregulated. Genetic studies have shown that BML is clonal in origin. Most BML lesions have been reported to be nonreactive to the antibody HMB-45, which recognize Pmel17, a melanosomal protein expressed in lung hamartomas, PEComas, and LAM; however, BML lesions may also show low reactivity.

Most patients with BML present with multiple pulmonary nodules, which usually develop after myomectomy or hysterectomy for the treatment of uterine fibroids. To date, there are no standard clinical criteria for diagnosing BML. In many cases, BML lung metastases are asymptomatic. Lung biopsies have been used to confirm the diagnosis of BML and rule out malignant tumors. For lymph node metastases of BML, as in our case, leiomyomatosis must be distinguished from several other spindle cell tumors involving the lymph nodes (primarily or secondary), such as Kaposi’s sarcoma, dendritic reticulum cell tumor, intranodal neurilemmoma, and metastatic spindle cell tumor.

Careful history taking and physical examination are useful to rule out many of the listed disorders, and another common differential diagnosis is pulmonary lymphangioleiomyomatosis (LAM). Pulmonary LAM is a rare slowly progressive lung disease that affects almost exclusively young women of reproductive age [35]. It is caused by mutations in the tuberous sclerosis genes [36] and characterized by cystic remodeling of the lung parenchyma. The most common clinical manifestations are progressive dyspnea on exertion, pneumothorax and chylous effusions. On the other hand, lack of systemic symptoms or no symptoms, along with a history of uterine fibroids or prior uterine surgery, is highly suggestive of BML. CT imaging of the chest and abdomen may suggest malignancy or infection. Ultimately, a histological diagnosis is required to rule out the diagnoses listed above and establish the diagnosis of BML. This may consist of lung biopsy and lesion resection.

Given the rarity of this disease, no standardized treatment for BML has been proposed. Several suggestions published in the literature include regular and careful observation, surgical resection with oophorectomy, progesterone therapy, and medical castration using aromatase inhibitors, estrogen receptor blockers (e.g., tamoxifen and raloxifene), tyrosine kinase inhibitors (e.g., imatinib), and GnRH agonists [37, 38]. Jennifer et al. [12] reported a case of pelvic leiomyoma with lung metastases in a patient who underwent bi-salpingo-oophorectomy and opted for HRT after surgery. Thoracic CT tomography showed an enlarged pulmonary nodule, and HRT was stopped. Letrozole was administered by a gynecologic oncologist for a slightly enlarged lung nodule on CT surveillance. Two years later, surveillance imaging confirmed the stability of the pulmonary BML, and the patient remained asymptomatic. This implies that the impact of estrogen on the tumor and HRT treatment should be carefully assessed before administration to the patient. Careful follow-up was also indicated. In our case, the patient underwent bi-oophorectomy and no special treatment was administered. Follow-up until now has been uneventful.

The reported prognosis of patients with BML is favorable. Pulmonary lesions are usually discovered several years (months to more than 30 years) after hysterectomy or myomectomy, and the growth rate of these tumors is slow. Although lung BML is rare and simultaneous metastases of the retroperitoneal lymph nodes after previous hysterectomy or myomectomy are even rarer, such patients are suggested to have prolonged surveillance and careful follow-up for early detection of recurrence or distant metastases. As therapeutic options are limited, new drugs or therapeutic strategies should be studied and considered. In addition, long-term follow-up may be useful in studying their implications in clinical practice.

## Data Availability

There was no dataset, as this was a case report. Patient data and details are available upon request by email.

## Abbreviations

BML: Benign metastasizing leiomyoma
MRI: Magnetic resonance imaging
PET: Positron emission tomography
HPF: High-power fields
FDG: Fluorodeoxyglucose
SUV: Standard uptake value
